# Ultrasonography and color Doppler in juvenile idiopathic arthritis: diagnosis and follow-up of ultrasound-guided steroid injection in the ankle region. A descriptive interventional study

**DOI:** 10.1186/1546-0096-9-4

**Published:** 2011-01-29

**Authors:** Louise Laurell, Michel Court-Payen, Susan Nielsen, Marek Zak, Mikael Boesen, Anders Fasth

**Affiliations:** 1 Department of Pediatrics, Skåne University Hospital, Lund University, Sweden; 2 Department of Diagnostic Imaging, Gildhøj Private Hospital, University of Copenhagen, Denmark; 3 Department of Pediatrics, Rigshospital, University of Copenhagen, Denmark; 4 Parker Institute, Frederiksberg Hospital, University of Copenhagen, Denmark; 5 Department of Pediatrics, University of Gothenburg, Sweden

## Abstract

**Background:**

The ankle region is frequently involved in juvenile idiopathic arthritis (JIA) but difficult to examine clinically due to its anatomical complexity. The aim of the study was to evaluate the role of ultrasonography (US) of the ankle and midfoot (ankle region) in JIA. Doppler-US detected synovial hypertrophy, effusion and hyperemia and US was used for guidance of steroid injection and to assess treatment efficacy.

**Methods:**

Forty swollen ankles regions were studied in 30 patients (median age 6.5 years, range 1-16 years) with JIA. All patients were assessed clinically, by US (synovial hypertrophy, effusion) and by color Doppler (synovial hyperemia) before and 4 weeks after US-guided steroid injection.

**Results:**

US detected 121 compartments with active disease (joints, tendon sheaths and 1 ganglion cyst). Multiple compartments were involved in 80% of the ankle regions. The talo-crural joint, posterior subtalar joint, midfoot joints and tendon sheaths were affected in 78%, 65%, 30% and 55% respectively. Fifty active tendon sheaths were detected, and multiple tendons were involved in 12 of the ankles. US-guidance allowed accurate placement of the corticosteroid in all 85 injected compartments, with a low rate of subcutaneous atrophy (4,7%). Normalization or regression of synovial hypertrophy was obtained in 89%, and normalization of synovial hyperemia in 89%. Clinical resolution of active arthritis was noted in 72% of the ankles.

**Conclusions:**

US enabled exact anatomical location of synovial inflammation in the ankle region of JIA patients. The talo-crural joint was not always involved. Disease was frequently found in compartments difficult to evaluate clinically. US enabled exact guidance of steroid injections, gave a low rate of subcutaneous atrophy and was proved valuable for follow-up examinations. Normalization or regression of synovial hypertrophy and hyperemia was achieved in most cases, which supports the notion that US is an important tool in the management of ankle involvement in JIA.

## Background

The ankle region (ankle and midfoot) is frequently diseased in juvenile idiopathic arthritis (JIA) [1,2] but the anatomical complexity of this area makes it difficult to evaluate clinically which of the numerous joints and surrounding tendons are involved in the inflammatory process. Several publications in adult rheumatology have shown high sensitivity of ultrasonography (US) for early detection of synovitis in various joints including the ankle [3-11], but there are only few reports dealing with pediatric rheumatology [12-14].

Intra-articular steroid injection is a treatment option for JIA patients with mono- and oligoarticular involvement or whose joints remain active during systemic treatment [15]. The clinical response to a palpation-guided intra-articular steroid injection is however poorer in the ankle than in other joints [16,17], which may be due to the anatomical complexity of this region preventing accurate placement of the needle tip.

In the present study, we investigated the usefulness of US of the ankle region in children with JIA for detection of synovial hypertrophy and hyperemia, for guidance of steroid injection and for assessment of treatment efficacy.

## Methods

The study was conducted over a period of 2.5 years at the Department of Pediatrics (Rigshospital) of the University of Copenhagen, Denmark. Forty ankle regions were investigated in 30 JIA patients with active disease, as judged by the treating physician. Twenty-one patients were female (70%), 9 male (30%) and the median age of the patients was 6.5 years (range 1-16). Eleven of the children had poly-JIA (median age 10 years), and 19 oligo-JIA (median age 5 years). Demographic features, clinical and laboratory assessment are listed in Table 1. The study was approved by the local research ethics committee (Videnskabsetiske Komiteer for Region Hovedstaden), and all parents gave informed consent for the participation of their children.

**Table 1:** Clinical and laboratory assessment in 30 JIA patients with 40 symptomatic ankles*

Characteristic	Number (%)	Median	Range
Sex			
Male	9 (30%)		
Female	21 (70%)		
Subgroups			
RF-negative polyarthritis	11 (37%)		
Oligoarthritis extended	6 (20%)		
Oligoarthritis persistent	13 (43%)		
Other subgroups	0		
Age at injection, years		6.5	1-16
Disease duration, years		2.0	0.5-13.9
Number of joints (n = 40) with			
Swelling	38 (95%)		
Pain	35 (88%)		
Tenderness at palpation	39 (98%)		
Limited range of motion	34 (85%)		
Active arthritis	40 (100%)		
VAS ankle pain patient/parent, cm		3.5	0.2-10.0
VAS global assessment patient/parent, cm		0.6	0-9.7
VAS global assessment physician, cm		2.8	1-6.8
CRP level, mg/l		2	0-22
ESR, mm/hour		14	8-69
HLA B27 positive, number of patients	5 (17%)		
ANA positive, number of patients	14 (47%)		
Second-line drug therapy			
Methotrexate	15 (50%)		
Biologic therapies			
Etanercept	3 (10%)		
Adalimumab	2 (7%)		
Abatacept	1 (3%)		
Systemic corticosteroid therapy	5 (17%)		

* RF = rheumatoid factor; VAS = visual analog scale; CRP = C-reactive protein; ESR = erythrocyte sedimentation rate; HLA = human leukocyte antigen; ANA = antinuclear antibodies

During the study period, an initial Doppler-US examination was performed (week 0) in all consecutive JIA patients with a clinically diseased ankle region. US revealed signs of synovitis in a total of 36 patients, 10 with involvement of both ankle regions. Six patients were excluded due to non-participation at follow-up, leaving 30 patients and 40 ankle regions for evaluation. At inclusion 26 patients had ongoing systemic treatment, 15 with methotrexate, 6 with methotrexate and biologics (3 etanercept, 2 adalimumab and 1 abatacept), and 5 with systemic corticosteroids. Four patients received an intra-articular steroid injection within the previous 3 months, but not in the same extremity. The pediatric rheumatologist and the radiologist made consensus decisions based on both clinical complaints and imaging results regarding which compartments to inject.

### Clinical and US assessment

Patients diagnosed with JIA, based on the revised criteria of the International League of Associations for Rheumatology (ILAR, 2004) [18], were examined by one of two experienced pediatric rheumatologists for clinical signs of involvement of the ankle region. Recorded clinical variables were: swelling, pain assessed by the patient/parent (Visual Analogue Scale, VAS), tenderness at palpation and limitation in the range of motion.

Patients with clinically active arthritis (swelling or limited range of motion with pain or tenderness) were assessed with US on the same day. The US examiner was a radiologist specialized in musculoskeletal US using a GE Logiq 9 scanner (General Electric Corporation, USA) equipped with a 16-4 MHz linear transducer (4D16L). The following joints and tendon sheaths were examined: anterior, anteromedial and anterolateral talo-crural joint (anterior, anteromedial and anterolateral recesses), posterior subtalar joint (lateral recess), anterior subtalar joint (dorsal and medial recesses), tibialis posterior, flexor digitorum longus, flexor hallucis longus, peroneus, tibialis anterior, extensor hallucis longus, and extensor digitorum longus. For each of these compartments the following US signs of disease were registered: synovial hypertrophy (presence/absence) and joint effusion (presence/absence) according to the OMERACT 7 (Outcome Measures in Rheumatology) definitions for ultrasonographic pathology in inflammatory arthritis [19]. The grade of synovial hyperemia was assessed by a semi-quantitative grading of color Doppler flow, where 0 indicates absence of flow and 1-3 indicates hyperemia. In the talo-crural joint, the synovial thickness of the anterior recess was also measured at 4 locations (proximal and distal on the midline, anterior to medial malleolus, and anterior to lateral malleolus). A mean synovial thickness (MST) was calculated from the 4 measurements at each occasion. The MST values before and after steroid injection were compared with paired t-test. Bone or cartilage erosions were registered if present.

### US-guided steroid injection

**Figure 1:**
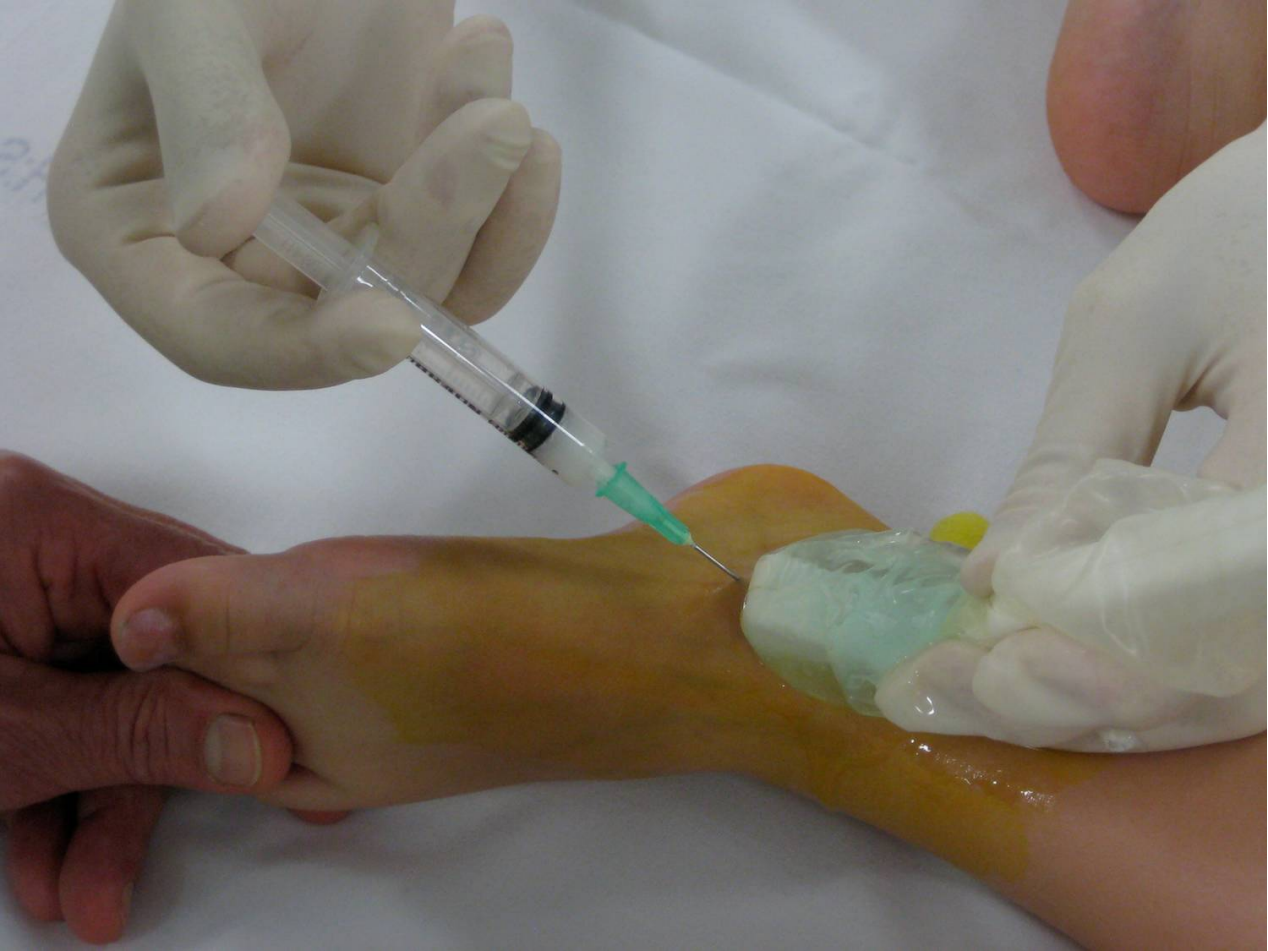
**US-guided steroid injection of the talo-crural joint **. The needle is inserted obliquely into the antero-medial recess of the joint (longitudinal US plane).

**Figure 2:**
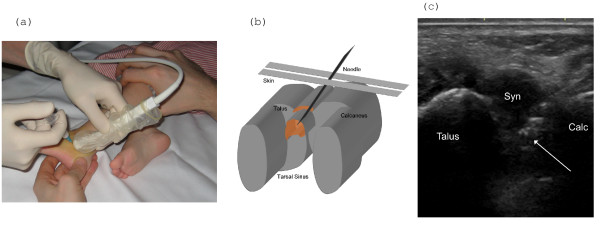
**US-guided injection in the antero-lateral recess of the posterior subtalar joint **. (A-B) Lateral oblique longitudinal scanning plane at the level of the posterior tarsal sinus. (C) The tip of the needle (arrow) is seen in the enlarged hypoechoic antero-lateral recess (Syn) which is bulging into the hyperechoic fat of the tarsal sinus, between talus and calcaneus (Calc).

For all US-guided injections triamcinolone acetonide 40 mg/ml was used according to local established practice. Dose estimation was based on the size of compartment to inject and the age of the child. Injections were performed by a free-hand technique using a 12-5 MHz linear hockey-stick transducer (i12L). The needle (21 G for joints, 23 G for tendon sheaths) was inserted along the US plane into joint recesses or tendons sheaths. The talo-crural joint was punctured anteromedially (Figure 1) or anterolaterally, the posterior subtalar joint anterolaterally (anterior to the peroneus tendons) - the anterolateral recess of the posterior subtalar joint was found from a coronal plane at the level of the tarsal sinus angling the transducer posteriorly (Figure 2). The talo-calcaneo-navicular joint was punctured dorsally or medially.

### Follow-up after injection

All patients underwent the same clinical and US assessment before (week 0) and at 4 weeks after the steroid injection. For the talo-crural joint, a decrease in MST of ≥80% was regarded as 'normalization' (inactive synovial tissue). A decrease of 20-80% was considered as 'regression'. For the other joints and tendons, only the presence (no treatment effect) or absence (normalization after treatment) of synovial hypertrophy was recorded. In the follow-up of synovial hyperemia, normalization was defined as total absence (grade 0) of color Doppler flow.

## Results

At week 0, Doppler US detected synovial hypertrophy, effusion, and/or hyperemia in 121 compartments (joints, tendon sheaths, and a ganglion cyst) (Table 2). Most ankles (n = 32) had involvement of multiple compartments. Synovial hypertrophy was found in 31 talo-crural joints (Table 2), and in 9 of those (29%) it was localized, involving only the lateral, anterior, or anterolateral aspect of the joint (2, 3, and 4 joints, respectively). Isolated talo-crural synovial hypertrophy was found in only 8% (3/40) of the ankles. The compartments most frequently involved in association with the talo-crural joint were the posterior subtalar joint (77%, 24/31) and tendon sheaths (48%, 15/31). Synovial hypertrophy was detected in 26 posterior subtalar joints (Table 2). Only 1 ankle showed isolated involvement of the posterior subtalar joint. In the 12 diseased midfoot areas, synovial hypertrophy was found in 10 talo-navicular joints, 2 naviculo-cuneiform I joints, and 1 tarso-metatarsal V joint (Table 2). Tendon involvement was found in 36 medial, 9 lateral and 5 anterior tendon sheaths. It was multiple in 12 ankles and bilateral in 2. Isolated tenosynovitis, without any joint involvement, was found in only 4 patients.

**Table 2:** US diagnosis of synovial hypertrophy and hyperemia in 40 ankles at week 0

Compartment	Number of ankles	Number of compartments with synovial hypertrophy (hyperemia)
Talo-crural joint	31 (78%)	31 (29)
Post-subtalar joint	26 (65%)	26 (25)
Midfoot joints	12 (30%)	13* (12)
Tendon sheaths	22 (55%)	50** (41)
Para-articular cyst	1 (3%)	1 (1)
All compartments		121 (108)

* One ankle with involvement of 2 anterior subtalar joints.

** Twelve ankles with involvement of multiple tendons.

Effusion was detected in 40% of the involved compartments, mostly tendon sheaths (33/50, 66%), but also 9 talo-crural joints (29%), 5 anterior subtalar joints (42%) and 1 para-articular cyst. Color Doppler examination showed synovial hyperemia in 108 of the 121 diseased compartments (89%, Table 2). Bone erosions were found in 3 patients, all in the talus. These subjects were 13-15 years old, 2 had polyarticular and 1 oligoarticular arthritis, with disease duration of 6, 102, and 129 months, respectively.

US-guided steroid injection was performed in 85 of the 121 diseased compartments, and this was done under general anesthesia in 26 patients (median age 5 years) and with nitrous oxide-oxygen analgesia in 4 patients (median age 15.5 years), according to local practice. Triamcinolone acetonide 40 mg/ml was used for all steroid injections. In joints, the injected dose was 40 mg in 83% (52/63) and 20 mg in the remaining 17%. In tendon sheaths (1 or 2 per patient), the dose was 20 mg in 86% (18/21) and 40 mg in the rest. A dose of 20 mg was injected into the cyst.

In 14 of the patients other joints (mostly the knee, but in some also the wrist or elbow) were injected with triamcinolone hexacetonide in the same session. The total steroid dose injected per patient was 40-340 mg (mean 100 mg, median 80 mg). During the study period, 12 ankles had 1 injection, and 28 had multiple injections (2-3 compartments in 25 ankles and 4-5 compartments in 3 ankles). The total time demanded for US-guided steroid injection including general anesthesia, was approximately 30 minutes. The time for injection was 5-15 minutes, depending on the number and sites of injections.

Ten patients had involvement of both ankle regions during the study period. In 5 of these patients bilateral symptoms occurred at the same time and both sides were injected in the same session. In 3 patients, new systemic therapies were started at the time of the steroid injection: 2 were previously untreated and were given methotrexate; the other patient had previously received abatacept, which was switched to etanercept.

### Result of US-guided steroid injection

**Figure 3:**
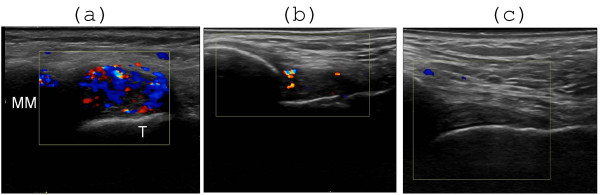
**Effect of US-guided steroid injection in the talo-crural joint as revealed by color Doppler **. (A) Before steroid injection there is synovial hyperemia anterior to the medial malleolus (MM). T = talus. (B) One week after steroid injection there is partial regression of hyperemia. (C) Normalization with complete regression of hyperemia 4 weeks after steroid injection.

US-guidance of the injection needle enabled real-time visualization of the procedure and quick and effective placement of the needle tip in all 85 of the treated compartments. Table 3 shows the effects on synovial hypertrophy detected in 85 injected and 36 non-injected compartments 4 weeks after treatment, and Table 4 presents the effects on synovial hyperemia in 81 injected and 27 non-injected compartments at the same time point. Considering talo-crural joints synovial hypertrophy was normalized in 55% (17/31) and regressed in 32% (10/31), and there was an overall effect of steroid injection in 87% (Table 3). Quantitative evaluation of MST of the talo-crural joint before and 4 weeks after steroid injection showed a statistically significant decrease (p < 0.001, paired t-test). Normalization of synovial hypertrophy was noted in 93% of the other compartments (Table 3). Normalization of hyperemia (grade 0) was seen in (72/81) 89% of injected compartments, partial regression in 8 compartments (10%) and persistent hyperemia (grade 2) in 1 compartment (1%) (Table 4, Figure 3). There was an equally good result for non-injected diseased compartments in most anatomical sites, except the posterior subtalar joints where synovial hypertrophy was normalized in only 60% and hyperemia in 50%.

**Table 3:** Effect on synovial hypertrophy in 121 compartments 4 weeks after US-guided steroid injection

	85 injected compartments		36 non-injected compartments	
	Normalization or regression	No effect	Normalization or regression	No effect
Talo-crural joints (31)	27/31 (87%)	4/31 (13%)	-	-
Post-subtalar joints (26)	20/21 (95%)	1/21 (5%)	3/5 (60%)	2/5 (40%)
Midfoot joints (13)	10/11 (91%)	1/11 (9%)	2/2 (100%)	0
Tendons (50)	18/21 (86%)	3/21 (14%)	28/29 (97%)	1/29 (3%)
Para-articular cyst (1)	1/1 (100%)	0	-	-
All compartments (121)	76/85 (89%)	9/85 (11%)	33/36 (92%)	3/36 (8%)

**Table 4:** Effect on hyperemia in 108 compartments 4 weeks after US-guided steroid injection

	81 injected compartments		27 non-injected compartments	
	Normalization	No normalization	Normalization	No normalization
Talo-crural joints (29)	25/29 (86%)	4/29 (14%)	-	-
Post-subtalar joints (25)	20/21 (95%)	1/21 (5%)	2/4 (50%)	2/4 (50%)
Midfoot joints (12)	8/10 (80%)	2/10 (20%)	2/2 (100%)	0
Tendons (41)	18/20 (90%)	2/20 (10%)	21/21 (100%)	0
Para-articular cyst (1)	1/1 (100%)	0	-	-
All compartments (108)	72/81 (89%)	9/81 (11%)	25/27 (93%)	2/27 (7%)

At clinical follow-up 4 weeks after steroid injection 29/40 ankles (72%) exhibited absence of active arthritis and 11/40 had partially improved. The range of motion was normalized in 29/40 ankles, partially improved in 9/40 and deteriorated in 2/40. Ankle pain totally regressed in 29/40 ankles, partially improved in 8/40 and deteriorated in 3/40. In 24/40 ankles (60%), all clinical parameters were completely normalized after 4 weeks.

### Relapses

Seven patients had relapse of symptoms and synovitis in the ankle region, verified by Doppler-US, during the 2.5-year study period. Two of these patients had a second relapse, and 1 had 3 relapses. All 7 patients were re-injected with US-guidance. In 4 of the 7 patients, local steroid injection was the only treatment given. Three of the patients were on second-line therapy (methotrexate) at the time of relapse, and in 1 patient methotrexate was added after the relapse. Relapse occurred in a previously injected compartment in 2 patients, in a different compartment only in 4 patients, and in both a previously injected and a different compartment in 1 patient. Relapses occurred after a mean of 6.3 months (median 6 months, range 4-11 months) and re-injections were performed within 1-2 weeks of relapse.

### Complications

Local subcutaneous atrophy was registered in 3 patients (2 two-year-olds and 1 nine-year-old) at 4 injection sites (1 talo-crural joint and 3 tendon sheaths), which represents a complication rate of 4.7% (4/85). No other complications were noted.

## Discussion

New disease-modifying therapy for JIA has changed the outcome and increased the need for imaging techniques more sensitive and specific than clinical examination alone. In three recent studies clinical examination and US findings of the ankle in children with JIA were compared, and clinical assessment was found to be inadequate in identifying the structures involved [12,14,20]. Several other studies have also shown US to be superior to clinical assessment for the detection of active arthritis, in children [21-26] and in adults [27,28]. Screening of JIA patients with US have revealed subclinical synovitis in 35-51%, leading to reclassification of patients as having polyarticular disease [12,20], which is in agreement with a previous report on RA [29].

US is suitable for examination of children of all ages and has certain advantages over MRI [30,31] being cheaper, mobile, instantly accessible bedside, easy to combine with clinical assessment (interactivity) and non-invasive. It does not require sedation, which facilitates repetitive examinations. Assessment of multiple locations is possible during the same session. Agitation is rarely a problem and young children can be seated in a parent's lap or play while being examined. Modern high-frequency US transducers provide unsurpassed resolution of the superficial musculoskeletal structures in children. An advantage of MRI is its ability to demonstrate bone marrow edema [32] a predictor of bone erosions in RA [33], and erosions in difficult-to-assess regions. Doppler-US adds further information by depicting articular and para-articular soft tissue hyperemia [10,12,34]. The Doppler signal can distinguish between active and inactive synovitis in RA, correlating to clinical and laboratory data [35], MRI [10] and histology [36]. Doppler-US is included in the ultrasonographic definitions of synovitis, tenosynovitis and enthesitis in adult rheumatology [19]. Different gradation systems of Doppler flow are employed, using quantitative [37] or semi-quantitative [10] methods. The latter is more frequently used in clinical practice [38] and the method used in our study. Hardly any data exists on the evaluation of synovial hyperemia by Doppler-US in JIA. Two investigations have demonstrated a correlation between Doppler flow and clinical activity [39,40].

Early involvement of the ankle region is common in JIA. In a Swedish population-based study 52% of patients with oligoarthritis and 22% with monoarthritis, respectively, had ankle region involvement at the time of diagnosis [2]. It is often assumed that ankle pain and swelling represent talo-crural synovitis, with the occasional exception of very obvious tendon involvement. Our results show the complex distribution of synovial involvement in multiple joints and tendon sheaths (Table 2), in concordance with 2 recent US investigations [12,13] and 2 MRI studies [17,41] of JIA patients.

The present study is a descriptive interventional study and was not designed to compare results from clinical and US assessments. In our investigation the talo-crural joint was involved in 78%, other studies report rates of 85% [17], 55% [41], 61% [13] and 67% [14] respectively. In our study the posterior subtalar joint was involved in 65%, compared to 40-77% in earlier reports [12,17,41]. Tendon sheaths were involved in 55% of the ankles in our study, compared to reports of 71 and 77% [13,41]. Tenosynovitis was isolated in 10% in our study, compared to 39% [13] and 5% [41]. Medial tendons were most frequently involved in our patients, in agreement with earlier findings [13]. All reports of frequent involvement of multiple compartments strongly suggest that imaging, MRI or more easily US [27], should be performed prior to ankle injections in children with JIA.

In US, synovial hypertrophy is detected as solid, non-compressible, hypoechoic tissue in connection to joint lines or surrounding tendons [19]. In children detection is more challenging than in adults as the synovial tissue often is difficult to distinguish from the hypoechoic cartilage of epiphyses. To avoid diagnostic errors, it is therefore important to have good knowledge of the age-dependent normal US appearance of each joint, and to use a meticulous scanning technique that allows clear interpretation of possible anisotropic artifacts.

The presence of juxta-articular flow at color Doppler examination in the growing child may either represent normal flow of the well-vascularised cartilage of the epiphysis or synovial hyperemia indicating inflammation. Flow in the cartilage is probably indicating normal cartilaginous flow in contrast to flow inside the synovium which probably indicates hyperemia.

In our study one third of the diseased talo-crural joints showed localized synovial hypertrophy with anterior, medial, or lateral localization. Other investigators using contrast-enhanced MRI have also found heterogeneous distribution of synovitis within joints [42]. Our US protocol did not include the posterior aspect of the talo-crural joint, and hence we cannot rule out any localized posterior synovitis.

The clinical effect of steroid injection into a joint or bursa depends on accurate placement of the needle tip in the affected cavity. Imaging-guided injections were found to give significantly better results than palpation-guided injections in adult arthritis/osteoarthritis in large and small joints [43-45], in patients with painful shoulders [46,47] and in children with JIA in the ankle region [17].

To our knowledge, our study is the first to report on US-guided steroid injection in the ankle region in JIA. Common clinical practice in JIA has been to perform a non-guided injection in the talo-crural joint when ankle swelling is present. Injection of the subtalar joint or tendon sheaths is less commonly performed, which might explain the poor outcome of steroid injections for ankle disease in JIA [12,16,17]. In our study, US showed no involvement of the talo-crural joint in 22% of cases, and involvement of other compartments in association with the talo-crural joint in 70%. The posterior subtalar joint was the second most frequently involved compartment in our study (65%). This joint is very difficult to inject without imaging-guidance in children [17,48,49].

Subcutaneous atrophy is a well-recognized adverse effect of intra-articular steroid injection in children, most likely to occur in small or complex joints such as the wrist or ankle in children under 4 years of age [50] or with a larger injection volume [49]. Using US-guidance in our study, the needle tip was always correctly localized before injection and possible extravasation of steroid into the subcutaneous tissue was detected immediately. To prevent reflux of liquid into the needle track the needle tip was inserted deeply into the superficially located tendon sheaths, and the needle was flushed with lidocaine before withdrawal. All injections were followed by thorough local compression. Despite these precautions, subcutaneous atrophy did occur in 3 patients. Two of those were very young, indicating that the volume of injected steroid might have mattered.

Follow-up of treatment efficacy of arthritis patients may be based on clinical examination and/or imaging. For the talo-crural and posterior subtalar joint clinical examination after steroid injection showed good results in 89% after 1-30 weeks (median 6 weeks, posterior subtalar joint only) [49], in 82% after 4 weeks [41], and in 67% after 6 months [17]. These studies are not directly comparable due to differences in design, number of patients, needle guidance technique (fluoroscopy, palpation) and follow-up time.

US follow-up after steroid injection [51] or other treatments [52] have been reported in several studies in adult rheumatology, but only in a few studies in children [21,24-26]. All JIA studies have focused on the knee and hip and none on the ankle. Systemic treatment with NSAID, DMARD, or corticosteroid [21,26] and intra-articular steroid injection [24,25] showed a decrease of effusion and synovial hypertrophy. Reduction in effusion occurred earlier than decrease in synovial hypertrophy [24,26].

**Figure 4:**
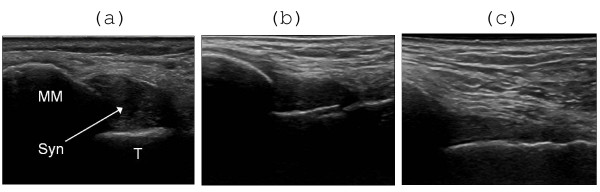
**Effect of US-guided steroid injection in the talo-crural joint **. (A) T = talus. Synovial thickening (Syn) anterior to the medial malleolus (MM), as shown by US before, (B) 1 week and (C) 4 weeks after steroid injection. There is regression of synovial hypertrophy without complete normalization.

**Figure 5:**
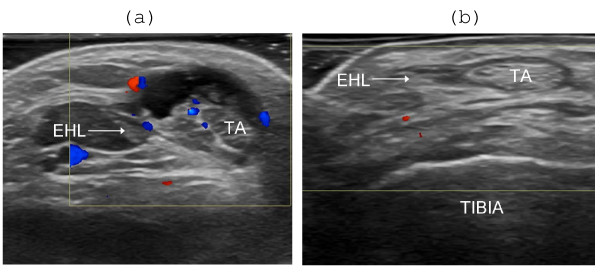
**Effect of US-guided steroid injection in the sheath of the tibialis anterior tendon **. (A) TA = tibialis anterior tendon, EHL = extensor hallucis longus tendon. Transversal scanning plane before steroid injection. Anechoic effusion, hyperechoic synovial hypertrophy and synovial hyperemia in the tendon sheath. (B) One week after injection there is complete regression of effusion, hypertrophy and hyperemia.

**Figure 6:**
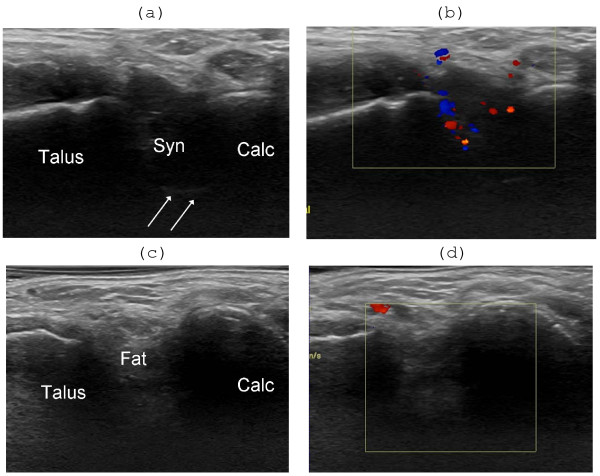
**Effect of US-guided steroid injection in the antero-lateral recess of the posterior subtalar joint **. Lateral oblique longitudinal scanning planes. (A) Before injection there is an enlarged hypoechoic antero-lateral recess (Syn), with distinct deep margins (arrows), bulging into the hyperechoic fat of the tarsal sinus between talus and calcaneus (Calc), and (B) synovial hyperemia at color Doppler examination. (C) One week after injection the recess is not visualized (D) and there is complete regression of synovial hyperemia.

Our study is the first reporting on US, and Doppler-US, for follow-up of steroid injections in the ankle region in JIA patients. We noted that effusion disappeared completely in the absolute majority of the injected compartments after 1 week. Table 3 illustrates the effect of US-guided steroid injection on synovial hypertrophy, with an overall normalization or regression of 89% at week 4, lowest for the talo-crural joint (87%, Figure 4), and the tendon sheaths (86%, Figure 5), and highest for other joints (91-95%, Figure 6). The somewhat lower result for the talo-crural joint might be explained by the different way we recorded synovial thickness in this particular joint. The large anterior recess of the talo-crural joint is easily accessible to US. Thus we evaluated the synovial membrane of that compartment in greater detail by measuring the thickness at 4 separate well-defined locations, summarizing the results as a single value (MST). This technique enabled detection of small areas of residual synovial tissue which is often seen in inactive synovitis [53,54]. We considered a residual synovial thickening of ≤20% at follow-up after steroid injection in the talo-crural joint to be normal. US synovial measurement was more challenging in the other smaller compartments i.e. the tarsal joints and tendon sheaths, and hence only the qualitative presence (lack of treatment effect) or absence (normalization) of synovial tissue was recorded.

Our finding of synovial hyperemia in almost all diseased compartments in the ankle region agrees with recent findings in JIA patients of hyperemia in 93% of symptomatic MCP-joints [39], but is higher than the 77% detected in symptomatic knees [40]. Doppler-US has become an important technique for follow-up of adult arthritis after steroid injections [55] or systemic anti-TNF [56]. In JIA only 2 studies have been published, prior to our present study, using Doppler-US for follow-up of systemic corticosteroid [40] and NSAID [34] treatment.

We also performed US follow-up of the 36 diseased compartments that did not receive steroid injections, which has not been considered in previous studies. In these compartments a high degree of normalization or regression of synovial hypertrophy and hyperemia was noted (Tables 3 and 4). An explanation for this beneficial effect may be systemic absorption of steroid or the presence of anatomical communications between adjacent injected and non-injected compartments. Arthrographic studies have shown communication between the talo-crural and the posterior subtalar joint in 4-16% of adults [57] but only in 0.5% of children [48]. Normally, there is no communication between the talo-crural joint and peroneal or tibial posterior sheaths, but the talo-crural joint may communicate with the flexor hallucis longus or the flexor digitorum longus sheath in 10-20% of adults [58] and 1% of children [48]. In our study all the tendons with persistent disease 4 weeks after steroid injection were of the communicating type.

Our US examination protocol did not include all compartments potentially causing symptoms in the ankle region e.g. the posterior talo-crural joint and plantar midfoot. A revised and more appropriate scanning protocol for juvenile arthritis that includes these structures as well will be used in the future.

## Conclusions

Our results highlight the value of US in pediatric rheumatology. US provided exact information of the anatomical location of inflamed structures in the ankle region. The talo-crural joint was not always involved and disease was frequently found in other compartments difficult to evaluate clinically (as the posterior subtalar joint). US enabled exact guidance of steroid injections with a low rate of subcutaneous atrophy, and was well suited for follow-up examinations. Normalization or regression of synovial hypertrophy and hyperemia was achieved in most cases, suggesting that US assessment prior to steroid injection, and US guidance of injections in this region would potentially improve treatment efficacy.

## Competing interests

The authors declare that they have no competing interests.

## Authors' contributions

LL participated in the design of the study, in the ultrasound and clinical examinations, in the acquisition of data, in the statistical analysis and was responsible for the analysis of results and for the draft of the manuscript.

MCP participated in the design of the study and in the analysis of results, performed the ultrasound examinations and helped to draft the manuscript.

SN participated in the design of the study, in performing the clinical examinations and in revising the manuscript.

MZ participated in the design of the study, in performing the clinical examinations and in revising the manuscript.

MB participated in the design of the study and in revising the manuscript.

AF participated in the design of the study, the analysis of results and in drafting the manuscript.

All authors read and approved the final manuscript.

## Authors' Information

Louise Laurell M.D. is a Consultant in Pediatrics at the Department of Pediatrics, Skåne University Hospital, Lund University, Sweden.

Michel Court-Payen M.D., Ph.D. is a Consultant in Radiology at the Department of Diagnostic Imaging, Gildhøj Private Hospital, University of Copenhagen, Denmark.

Susan Nielsen M.D. is a Consultant in Pediatrics, Department of Pediatrics, Rigshospital, University of Copenhagen, Denmark.

Marek Zak M.D. is a Consultant in Pediatrics at the Department of Pediatrics, Rigshospital, University of Copenhagen, Denmark.

Mikael Boesen M.D., Ph.D., is a Consultant in Radiology, Parker Institute, Frederiksberg Hospital, University of Copenhagen, Denmark.

Anders Fasth M.D., Ph.D. is a Consultant and Professor of Pediatric Immunology at the Department of Pediatrics, University of Gothenburg, Sweden.

## References

[B1] Flato B Lien G Smerdel A Vinje O Dale K Johnston V Sorskaar D Moum T Ploski R Forre O Prognostic factors in juvenile rheumatoid arthritis: a case-control study revealing early predictors and outcome after 14.9 years J Rheumatol 2003 30 386 393 12563700

[B2] Gare BA Fasth A The natural history of juvenile chronic arthritis: a population based cohort study. I. Onset and disease process J Rheumatol 1995 22 295 307 7738954

[B3] Weiner SM Jurenz S Uhl M Lange-Nolde A Warnatz K Peter HH Walker UA Ultrasonography in the assessment of peripheral joint involvement in psoriatic arthritis: a comparison with radiography, MRI and scintigraphy Clin Rheumatol 2008 27 983 989 10.1007/s10067-008-0835-y 18259687

[B4] Filippucci E Iagnocco A Meenagh G Riente L Delle Sedie A Bombardieri S Valesini G Grassi W Ultrasound imaging for the rheumatologist VII. Ultrasound imaging in rheumatoid arthritis Clin Exp Rheumatol 2007 25 5 10 17417983

[B5] Brown AK O'Connor PJ Roberts TE Wakefield RJ Karim Z Emery P Recommendations for musculoskeletal ultrasonography by rheumatologists: setting global standards for best practice by expert consensus Arthritis Rheum 2005 53 83 92 10.1002/art.20926 15696575

[B6] Scheel AK Schmidt WA Hermann KG Bruyn GA D'Agostino MA Grassi W Iagnocco A Koski JM Machold KP Naredo E Interobserver reliability of rheumatologists performing musculoskeletal ultrasonography: results from a EULAR "Train the trainers" course Ann Rheum Dis 2005 64 1043 1049 10.1136/ard.2004.030387 15640263 PMC1755572

[B7] Schmidt WA Schmidt H Schicke B Gromnica-Ihle E Standard reference values for musculoskeletal ultrasonography Ann Rheum Dis 2004 63 988 994 10.1136/ard.2003.015081 15249327 PMC1755091

[B8] Szkudlarek M Court-Payen M Jacobsen S Klarlund M Thomsen HS Ostergaard M Interobserver agreement in ultrasonography of the finger and toe joints in rheumatoid arthritis Arthritis Rheum 2003 48 955 962 10.1002/art.10877 12687537

[B9] Backhaus M Burmester GR Gerber T Grassi W Machold KP Swen WA Wakefield RJ Manger B Guidelines for musculoskeletal ultrasound in rheumatology Ann Rheum Dis 2001 60 641 649 10.1136/ard.60.7.641 11406516 PMC1753749

[B10] Szkudlarek M Court-Payen M Strandberg C Klarlund M Klausen T Ostergaard M Power Doppler ultrasonography for assessment of synovitis in the metacarpophalangeal joints of patients with rheumatoid arthritis: a comparison with dynamic magnetic resonance imaging Arthritis Rheum 2001 44 2018 2023 10.1002/1529-0131(200109)44:9<2018::AID-ART350>3.0.CO;2-C 11592362

[B11] Koski JM Ultrasonography of the subtalar and midtarsal joints J Rheumatol 1993 20 1753 1755 8295189

[B12] Magni-Manzoni S Epis O Ravelli A Klersy C Veisconti C Lanni S Muratore V Scire CA Rossi S Montecucco C Comparison of clinical versus ultrasound-determined synovitis in juvenile idiopathic arthritis Arthritis Rheum 2009 61 1497 1504 10.1002/art.24823 19877100

[B13] Rooney ME McAllister C Burns JF Ankle Disease in Juvenile Idiopathic Arthritis: Ultrasound Findings in Clinically Swollen Ankles J Rheumatol 2009 37 1725 1729 10.3899/jrheum.080508 19411390

[B14] Pascoli L Wright S McAllister C Rooney M Prospective Evaluation of Clinical and Ultrasound Findings in Ankle Disease in Juvenile Idiopathic Arthritis: Importance of Ankle Ultrasound J Rheumatol 2010 37 2409 2414 10.3899/jrheum.091262 20843904

[B15] Ravelli A Martini A Juvenile idiopathic arthritis Lancet 2007 369 767 778 10.1016/S0140-6736(07)60363-8 17336654

[B16] Marti P Molinari L Bolt IB Seger R Saurenmann RK Factors influencing the efficacy of intra-articular steroid injections in patients with juvenile idiopathic arthritis Eur J Pediatr 2008 167 425 430 10.1007/s00431-007-0525-9 17562077

[B17] Remedios D Martin K Kaplan G Mitchell R Woo P Rooney M Juvenile chronic arthritis: diagnosis and management of tibio-talar and sub-talar disease Br J Rheumatol 1997 36 1214 1217 10.1093/rheumatology/36.11.1214 9402868

[B18] Petty RE Southwood TR Manners P Baum J Glass DN Goldenberg J He X Maldonado-Cocco J Orozco-Alcala J Prieur AM International League of Associations for Rheumatology classification of juvenile idiopathic arthritis: second revision, Edmonton, 2001 J Rheumatol 2004 31 390 392 14760812

[B19] Wakefield RJ Balint PV Szkudlarek M Filippucci E Backhaus M D'Agostino MA Sanchez EN Iagnocco A Schmidt WA Bruyn GA Musculoskeletal ultrasound including definitions for ultrasonographic pathology J Rheumatol 2005 32 2485 2487 16331793

[B20] Haslam KE McCann LJ Wyatt S Wakefield RJ The detection of subclinical synovitis by ultrasound in oligoarticular juvenile idiopathic arthritis: a pilot study Rheumatology (Oxford) 2010 49 123 127 10.1093/rheumatology/kep339 19933594

[B21] Kakati P Sodhi KS Sandhu MS Singh S Katariya S Khandelwal N Clinical and ultrasound assessment of the knee in children with juvenile rheumatoid arthritis Indian J Pediatr 2007 74 831 836 10.1007/s12098-007-0148-1 17901669

[B22] El-Miedany YM Housny IH Mansour HM Mourad HG Mehanna AM Megeed MA Ultrasound versus MRI in the evaluation of juvenile idiopathic arthritis of the knee Joint Bone Spine 2001 68 222 230 10.1016/S1297-319X(01)00269-X 11394622

[B23] Gylys-Morin VM Graham TB Blebea JS Dardzinski BJ Laor T Johnson ND Oestreich AE Passo MH Knee in early juvenile rheumatoid arthritis: MR imaging findings Radiology 2001 220 696 706 10.1148/radiol.2203000461 11526269

[B24] Cellerini M Salti S Trapani S D'Elia G Falcini F Villari N Correlation between clinical and ultrasound assessment of the knee in children with mono-articular or pauci-articular juvenile rheumatoid arthritis Pediatr Radiol 1999 29 117 123 10.1007/s002470050554 9933332

[B25] Eich GF Halle F Hodler J Seger R Willi UV Juvenile chronic arthritis: imaging of the knees and hips before and after intraarticular steroid injection Pediatr Radiol 1994 24 558 563 10.1007/BF02012732 7724276

[B26] Sureda D Quiroga S Arnal C Boronat M Andreu J Casas L Juvenile rheumatoid arthritis of the knee: evaluation with US Radiology 1994 190 403 406 8284388 10.1148/radiology.190.2.8284388

[B27] d'Agostino MA Ayral X Baron G Ravaud P Breban M Dougados M Impact of ultrasound imaging on local corticosteroid injections of symptomatic ankle, hind-, and mid-foot in chronic inflammatory diseases Arthritis Rheum 2005 53 284 292 15818652 10.1002/art.21078

[B28] Szkudlarek M Narvestad E Klarlund M Court-Payen M Thomsen HS Ostergaard M Ultrasonography of the metatarsophalangeal joints in rheumatoid arthritis: comparison with magnetic resonance imaging, conventional radiography, and clinical examination Arthritis Rheum 2004 50 2103 2112 10.1002/art.20333 15248207

[B29] Wakefield RJ Green MJ Marzo-Ortega H Conaghan PG Gibbon WW McGonagle D Proudman S Emery P Should oligoarthritis be reclassified? Ultrasound reveals a high prevalence of subclinical disease Ann Rheum Dis 2004 63 382 385 10.1136/ard.2003.007062 15020331 PMC1754934

[B30] Damasio MB Malattia C Martini A Toma P Synovial and inflammatory diseases in childhood: role of new imaging modalities in the assessment of patients with juvenile idiopathic arthritis Pediatr Radiol 2010 40 985 998 10.1007/s00247-010-1612-z 20432018

[B31] McKay GM Cox LA Long BW Imaging juvenile idiopathic arthritis: assessing the modalities Radiol Technol 2010 81 318 327 20207788

[B32] Jimenez-Boj E Nobauer-Huhmann I Hanslik-Schnabel B Dorotka R Wanivenhaus AH Kainberger F Trattnig S Axmann R Tsuji W Hermann S Bone erosions and bone marrow edema as defined by magnetic resonance imaging reflect true bone marrow inflammation in rheumatoid arthritis Arthritis Rheum 2007 56 1118 1124 10.1002/art.22496 17393390

[B33] McQueen FM Benton N Perry D Crabbe J Robinson E Yeoman S McLean L Stewart N Bone edema scored on magnetic resonance imaging scans of the dominant carpus at presentation predicts radiographic joint damage of the hands and feet six years later in patients with rheumatoid arthritis Arthritis Rheum 2003 48 1814 1827 10.1002/art.11162 12847674

[B34] Shanmugavel C Sodhi KS Sandhu MS Sidhu R Singh S Katariya S Khandelwal N Role of Power Doppler sonography in evaluation of therapeutic response of the knee in juvenile rheumatoid arthritis Rheumatol Int 2008 28 573 578 10.1007/s00296-007-0482-7 17987293

[B35] Naredo E Bonilla G Gamero F Uson J Carmona L Laffon A Assessment of inflammatory activity in rheumatoid arthritis: a comparative study of clinical evaluation with grey scale and power Doppler ultrasonography Ann Rheum Dis 2005 64 375 381 10.1136/ard.2004.023929 15708891 PMC1755396

[B36] Walther M Harms H Krenn V Radke S Faehndrich TP Gohlke F Correlation of power Doppler sonography with vascularity of the synovial tissue of the knee joint in patients with osteoarthritis and rheumatoid arthritis Arthritis Rheum 2001 44 331 338 10.1002/1529-0131(200102)44:2<331::AID-ANR50>3.0.CO;2-0 11229463

[B37] Hau M Schultz H Tony HP Keberle M Jahns R Haerten R Jenett M Evaluation of pannus and vascularization of the metacarpophalangeal and proximal interphalangeal joints in rheumatoid arthritis by high-resolution ultrasound (multidimensional linear array) Arthritis Rheum 1999 42 2303 2308 10.1002/1529-0131(199911)42:11<2303::AID-ANR7>3.0.CO;2-4 10555024

[B38] Schmidt WA Technology Insight: the role of color and power Doppler ultrasonography in rheumatology Nature Clinical Practice Rheumatology 2007 3 35 42 10.1038/ncprheum0377 17203007

[B39] Karmazyn B Bowyer SL Schmidt KM Ballinger SH Buckwalter K Beam TT Ying J US findings of metacarpophalangeal joints in children with idiopathic juvenile arthritis Pediatr Radiol 2007 37 475 482 10.1007/s00247-007-0438-9 17415601

[B40] Shahin AA el-Mofty SA el-Sheikh EA Hafez HA Ragab OM Power Doppler sonography in the evaluation and follow-up of knee involvement in patients with juvenile idiopathic arthritis Z Rheumatol 2001 60 148 155 10.1007/s003930170063 11475602

[B41] Tynjala P Honkanen V Lahdenne P Intra-articular steroids in radiologically confirmed tarsal and hip synovitis of juvenile idiopathic arthritis Clin Exp Rheumatol 2004 22 643 648 15485022

[B42] Ostergaard M Stoltenberg M Henriksen O Lorenzen I Quantitative assessment of synovial inflammation by dynamic gadolinium-enhanced magnetic resonance imaging. A study of the effect of intra-articular methylprednisolone on the rate of early synovial enhancement Br J Rheumatol 1996 35 50 59 10.1093/rheumatology/35.1.50 8624624

[B43] Sibbitt WL Jr Peisajovich A Michael AA Park KS Sibbitt RR Band PA Bankhurst AD Does sonographic needle guidance affect the clinical outcome of intraarticular injections? J Rheumatol 2009 36 1892 1902 10.3899/jrheum.090013 19648304

[B44] Jones A Regan M Ledingham J Pattrick M Manhire A Doherty M Importance of placement of intra-articular steroid injections Bmj 1993 307 1329 1330 10.1136/bmj.307.6915.1329 8257889 PMC1679404

[B45] Peetrons P Court-Payen M Gangi A, Guth S, Guermazi A Ultrasound-Guided Musculoskeletal Interventional Procedures Imaging in Percutaneous Musculoskeletal Interventions 2008 Springer 385 398

[B46] Naredo E Cabero F Beneyto P Cruz A Mondejar B Uson J Palop MJ Crespo M A randomized comparative study of short term response to blind injection versus sonographic-guided injection of local corticosteroids in patients with painful shoulder J Rheumatol 2004 31 308 314 14760802

[B47] Eustace JA Brophy DP Gibney RP Bresnihan B FitzGerald O Comparison of the accuracy of steroid placement with clinical outcome in patients with shoulder symptoms Ann Rheum Dis 1997 56 59 63 10.1136/ard.56.1.59 9059143 PMC1752250

[B48] Sahlstedt Simultaneous arthrography of the talocrural and talonavicular joints in children. I. Technique Acta Radiol Diagn (Stockh) 1976 17 545 556 983756 10.1177/028418517601705a01

[B49] Beukelman T Arabshahi B Cahill AM Kaye RD Cron RQ Benefit of intraarticular corticosteroid injection under fluoroscopic guidance for subtalar arthritis in juvenile idiopathic arthritis J Rheumatol 2006 33 2330 2336 16981290

[B50] Job-Deslandre C Menkes CJ Complications of intra-articular injections of triamcinolone hexacetonide in chronic arthritis in children Clin Exp Rheumatol 1990 8 413 416 2397629

[B51] Terslev L Torp-Pedersen S Qvistgaard E Danneskiold-Samsoe B Bliddal H Estimation of inflammation by Doppler ultrasound: quantitative changes after intra-articular treatment in rheumatoid arthritis Ann Rheum Dis 2003 62 1049 1053 10.1136/ard.62.11.1049 14583566 PMC1754363

[B52] Ziswiler HR Aeberli D Villiger PM Moller B High-resolution ultrasound confirms reduced synovial hyperplasia following rituximab treatment in rheumatoid arthritis Rheumatology (Oxford) 2009 48 939 943 10.1093/rheumatology/kep139 19491302

[B53] Brown AK Quinn MA Karim Z Conaghan PG Peterfy CG Hensor E Wakefield RJ O'Connor PJ Emery P Presence of significant synovitis in rheumatoid arthritis patients with disease-modifying antirheumatic drug-induced clinical remission: evidence from an imaging study may explain structural progression Arthritis Rheum 2006 54 3761 3773 10.1002/art.22190 17133543

[B54] Frosch M Foell D Ganser G Roth J Arthrosonography of hip and knee joints in the follow up of juvenile rheumatoid arthritis Ann Rheum Dis 2003 62 242 244 10.1136/ard.62.3.242 12594110 PMC1754460

[B55] Filippucci E Farina A Carotti M Salaffi F Grassi W Grey scale and power Doppler sonographic changes induced by intra-articular steroid injection treatment Ann Rheum Dis 2004 63 740 743 10.1136/ard.2003.007971 15140784 PMC1755043

[B56] Naredo E Moller I Cruz A Carmona L Garrido J Power Doppler ultrasonographic monitoring of response to anti-tumor necrosis factor therapy in patients with rheumatoid arthritis Arthritis Rheum 2008 58 2248 2256 10.1002/art.23682 18668537

[B57] Trnka HJ Ivanic G Trattnig S Arthrography of the foot and ankle. Ankle and subtalar joint Foot Ankle Clin 2000 5 49 62 vi 11232081

[B58] Cassar-Pullicino VN Tins B Verlag S Anatomy, arthrography, Bursography and Tenography of the Ankle and Footimaging of the Foot and Ankle 2003 27 42

